# Prophylactic placement of inferior vena cava filters and the risk of death or venous thromboembolism in severe trauma patients: a retrospective study comparing two hospitals with different approaches

**DOI:** 10.1177/2058460121999345

**Published:** 2021-03-09

**Authors:** Thien Trung Tran, Haraldur Bjarnason, Jennifer McDonald, Nils Oddvar Skaga, Damon E Houghton, Brian Kim, Knut Stavem, Nils Einar Kløw

**Affiliations:** 1 Institute of Clinical Medicine, Faculty of Medicine, University of Oslo, Oslo, Norway; 2 Department of Diagnostic Imaging and Intervention, Akershus University Hospital, Lørenskog, Norway; 3 Department of Radiology, Mayo Clinic, Rochester, MN, USA; 4 Department of Anesthesiology, Oslo University Hospital Ullevål, Oslo, Norway; 5 Department of Hematology, Mayo Clinic, Rochester, MN, USA; 6 Division of Trauma, Critical Care and General Surgery, Mayo Clinic, Rochester, MN, USA; 7 Department of Pulmonary Medicine, Akershus University Hospital, Lørenskog, Norway; 8 Health Services Research Department (HØKH), Akershus University Hospital, Lørenskog, Norway; 9 Division of Radiology and Nuclear Medicine, Oslo University Hospital Ullevål, Oslo, Norway

**Keywords:** Trauma, embolism/thrombosis, vena cava, filter insertions, outcomes analysis, comparative studies

## Abstract

**Background:**

Prophylactic use of inferior vena cava filters to prevent pulmonary embolism in trauma is controversial. The practice varies between hospitals and countries, in part due to conflicting evidence and guidelines.

**Purpose:**

To compare the effects of pulmonary embolism, deep venous thrombosis and mortality in two hospitals using prophylactic inferior vena cava filter placement or prophylactic anticoagulation alone.

**Material and Methods:**

Patients presenting with severe trauma were recruited from two level-1 trauma centres between January 2008 and December 2013. Recruited patients from an US hospital having prophylactic inferior vena cava filter inserted were compared to a Scandinavian hospital using prophylactic anticoagulation alone. Inclusion criteria were age >15 years, Injury Severity Score >15 and survival >24 h after hospital admission. Patients with venous thromboembolism diagnosed prior to inferior vena cava filter placement were excluded. A Cox proportional hazard regression model was used with adjustment for immortal time bias and predictor variables.

**Results:**

In total, 951 patients were reviewed, 282 from an US hospital having inferior vena cava filters placed and 669 from a Scandinavian hospital without inferior vena cava filters. The mean age was 45.9 vs. 47.4 years and the mean Injury Severity Score was 29.8 vs. 25.9, respectively. Inferior vena cava filter placement was not associated with the hazard of pulmonary embolism (Hazard ratio=0.43; 95% confidence interval (CI) 0.12, 1.45; P=0.17) or mortality (Hazard ratio=1.16; 95% CI 0.70, 1.95; P=0.56). However, an increased rate of deep venous thrombosis was observed with inferior vena cava filters in place (Hazard ratio=3.75; 95% CI 1.68, 8.36; P=0.001).

**Conclusion:**

In severely injured trauma patients, prophylactic inferior vena cava filter placement was not associated with pulmonary embolism or mortality. However, inferior vena cava filters were associated with increased rate of deep venous thrombosis.

## Introduction

Severely injured patients are at risk of both venous thromboembolism (VTE) and trauma-related haemorrhages that may lead to death. The risk of VTE, which encompasses deep venous thrombosis (DVT) and pulmonary embolism (PE), is associated with the severity and certain types of traumas, as well as the length of immobility.^1^ VTE is an important cause of preventable morbidity and mortality in this group of patients, and pharmacological prophylaxis with unfractionated or low-molecular weight heparin is recommended to reduce the risk of VTE.^2^

In patients without a recent history of VTE, placement of inferior vena cava (IVC) filters is controversial due to conflicting evidence.^3–5^ Observational studies have reported that IVC filters lower the risk of PE and mortality, but many of these studies may suffer from immortal time bias and selection bias, in particular in cohort studies.^6,7^ These biases may explain the differences between these observational studies and a recent randomized trial, which did not show a benefit of prophylactic IVC filters to reduce the risk of PE or death.^8^

In this retrospective study, we compared two hospitals with different practices in management of trauma patients to determine if placement of IVC filters in one hospital alters the risk of PE, DVT and mortality when accounting for immortal time bias and selection bias.

## Material and Methods

### Study design and setting

This is an observational registry study of trauma patients admitted to one hospital in the US and one in Scandinavia, both level-1 trauma centres. Data were recorded prospectively in the two hospitals’ trauma registries. About 2500 and 2000 patients were enrolled in each hospital’s trauma registry per year, respectively, and blunt trauma accounted for more than 90% of the admissions in both centres. Approximately 20% of the patients with an Injury Severity Score (ISS) >15 received an IVC filter at the US hospital, but none were considered for or had an IVC filter placed at the Scandinavian hospital.^9^

### Patient inclusion and exclusion

Patients were included between January 2008 and December 2013 from Mayo Clinic, Rochester, MN, USA, and between January 2009 and December 2012 from Oslo University Hospital Ullevål, Oslo, Norway. Inclusion criteria were the same for both hospitals: age >15 years, ISS >15 upon admission, the Abbreviated Injury Scale (AIS) severity score >2 upon admission for either head/chest/abdomen/long bones/pelvis/spine alone or in combination, and survival >24 h after hospital admission.^10^

At the Mayo Clinic, all eligible patients having prophylactic IVC filter placement during the period were included. Computational extraction protocols according to the inclusion and exclusion criteria were used to find the patients in the trauma registry databases. However, patients with VTE diagnosed prior to IVC filter placement were excluded.

At Oslo University Hospital Ullevål, in order to reduce the workload with verification of end-points, we chose to allocate half of the patients to the control group. The patients were chosen by selecting every alternate patient among consecutive eligible patients in the trauma registry during the period. This would give approximately a 1:2 ratio of IVC filter patients to controls, which should ensure acceptable statistical power in the analysis.

### Thromboembolic end-points and verification

At Mayo Clinic, thromboembolic events identified in the trauma registry were crosschecked with medical records in order to validate findings. This was done either automatically with computational extraction algorithms (demographics, ICD-9 codes, Current-Procedural-Terminology-Codes) or by free text search of medical records.^11–13^ All positive endpoints identified by the trauma registry were confirmed by physician chart review.

At Oslo University Hospital Ullevål, the identification and verification of thromboembolic events were done manually by a physician, who reviewed and cross-checked medical records in the computerized radiological information system, picture archiving and communication system and patient record system in patients initially identified in the hospital-based trauma registry. In both institutions, PE was diagnosed by computed tomography (CT) pulmonary angiography. DVT in the lower extremities were diagnosed by either duplex ultrasound or CT venography. Referral to diagnostic imaging was based on clinical suspicion.

### Mortality verification

At Mayo Clinic, all-cause mortality was ascertained via link to the hospital’s records for deaths. At Oslo University Hospital Ullevål, mortality was ascertained by linkage to the National Population Register. Deaths after hospital discharge were not used in the analysis.

### Ethical approval

The study was approved by both participating institutions in accordance with the respective local regulations governing clinical research.

### Statistical analysis

Patient characteristics and crude outcomes are presented using the mean (standard deviation) or number (%), as appropriate. Groups were compared using the t-test or chi-squared test. The association of IVC filter placement with in-hospital PE, DVT and mortality were analysed using a Cox proportional hazards regression model, with covariates included for adjustment as presented in the footnotes of Table 3. IVC filter status was entered as an independent variable in the model.

To adjust for immortal time bias, we used the Landmark approach.^14^ Using this approach, exposure status is determined for all patients at a predefined time point.^15^ For this analysis, we chose a prespecified time point of day 3 after injury. Therefore, group allocation was defined by IVC filter insertion status at day 3 or earlier, and outcomes (PE, DVT mortality) were only considered if occurring between the landmark and hospital discharge. In the Landmark method, only patients that are still alive or not discharged from the hospital at the landmark time are included in the analysis. Patients discharged from the hospital or dead before or at the landmark were excluded from the analyses. The patients were divided into two categories according to whether they had received an intervention (IVC filter) up to that time, and all interventions after the landmark time were ignored. A sensitivity analysis was performed with two different landmarks (day 2 and day 3 after injury) and when person-time before IVC filter placement was excluded; the latter approach was included to illustrate that this type of analysis may be more prone to bias.

Multiple imputation was used to fill in missing data on baseline characteristics for pulse rate (7% missing values), systolic blood pressure (6%), oxygen saturation (16%) and Glasgow Coma Scale (GCS) score (1%).

Stata 15.1 (StataCorp, College Station, TX, USA) was used for statistical analysis.

## Results

### Study population and interventions

In total, 951 patients were included, 669 from Oslo University Hospital Ullevål had no IVC filter placed and 282 from Mayo Clinic had an IVC filter placed. Baseline patient characteristics of the study population are presented in Table 1. There was a male predominance in both cohorts (73% vs. 67%). Mean age was similar in both cohorts (47.4 vs. 45.9 years, P=0.30). GCS score and maximum head AIS severity scores were higher in the non-filter cohort, while ISS and maximum AIS severity scores of chest, abdomen, bones and spines were higher in the IVC filter cohort.

**Table 1.** Baseline patient characteristics, mean (SD), unless specified otherwise.

Characteristics	Overall (n=951)	Non-IVC filter (n=669)	IVC filter (n=282)	P-value
Age	47.0 (20.9)	47.4 (20.7)	45.9 (21.4)	0.30
Male sex, number (%)	675 (71.0)	487 (72.8)	188 (67)	0.06
Injury severity score	27.1 (9.9)	25.9 (9.4)	29.8 (10.6)	<0.001
Median (range)	26 (16–75)	25 (16–75)	27 (16–75)	NA
Glasgow Coma Scale	11.1 (4.7)	11.8 (4.2)	9.7 (5.6)	<0.001
Median (range)	14 (3–15)	14 (3–15)	13 (3–15)	NA
Max head AIS severity score	2.9 (1.9)	3.0 (2.0)	2.5 (1.7)	<0.001
Max chest AIS severity score	1.8 (1.7)	1.5 (1.8)	2.3 (1.4)	<0.001
Max abdomen AIS severity score	1.0 (1.6)	0.9 (1.6)	1.3 (1.5)	<0.001
Max long bones/pelvis AIS severity score	1.2 (1.5)	0.9 (1.4)	1.9 (1.5)	<0.001
Max spine AIS severity score	1.3 (1.6)	1.0 (1.5)	1.9 (1.5)	<0.001
Transferred between hospitals, number (%)	509 (53.5)	360 (53.8)	149 (53)	0.78
Pulse rate, min^–1^	90.7 (22.8)	87.0 (20.0)	99 (26.1)	<0.001
Systolic blood pressure, mmHg	125.4 (29.9)	128.1 (29.9)	119.4 (29.0)	<0.001
Oxygen saturation (%)	97.2 (4.6)	97.4 (4.0)	97.0 (5.8)	0.27

IVC filter: inferior vena cava filter; Max: maximum; AIS: Abbreviated Injury Scale; NA: not applicable.

Pharmacological thromboprophylaxis during hospitalization was given to both cohorts (66.4% vs. 68.8%, P=0.47). The most common pharmacological thromboprophylaxis being used were low-molecular-weight heparin (Dalteparin, Enoxaparin) and unfractionated heparin. In patients where an IVC filter was placed, the insertion rate was 72% at day 2 or earlier and 84% at day 3 or earlier. Median time from injury to IVC filter placement was two days (range, 0–21 days). The median length of hospital stay was 15 days (2–148 days) for the IVC filter cohort and 8 days (0–83 days) for the non-IVC filter cohort.

### Venous thromboembolism events and deaths

The crude number of PE was 8 (1.2%) in the non-IVC filter vs. 5 (1.8%) in the IVC filter cohort (Table 2). The number of DVT was 4 (0.6%) in the non-IVC filter vs. 17 (6%) in the IVC filter cohort. Median time from injury to VTE (combined PE and DVT) was 13 days (range, 0–55 days) in the non-IVC filter cohort and 11 days (5–59 days) in the IVC filter cohort. The number of deaths was 55 (8.2%) in the non-IVC filter vs. 22 (7.8%) in the IVC filter cohort. The median time from injury to death was three days (range, 1–50 days) in the non-IVC filter cohort and seven days (range, 2–16 days) in the IVC filter cohort.

**Table 2.** In-hospital outcomes, crude numbers (%).

	Non-IVC filter (n=669)	IVC filter (n=282)
Pulmonary embolism	8 (1.2)	5 (1.8)
Deep venous thrombosis	4 (0.6)	17 (6.0)
Mortality	55 (8.2)	22 (7.8)

IVC filter: inferior vena cava filter.

**Table 3.** Analysis from landmark to events (death, PE, DVT) or discharge.

	Unadjusted			Adjusted		
	Death	Pulmonary embolism	Deep venous thrombosis	Death*	Pulmonaryembolism**	Deep venous thrombosis **
All patients#						
No. of patients, IVC filter/non-filter	282/669	282/669	282/669	282/669	282/669	282/669
No. of events, IVC filter/non-filter	22/55	5/8	17/4	22/55	5/8	17/4
Hazard ratio	0.92	3.98	11.80	0.54	3.11	10.95
95% CI	0.63, 1.34	1.68, 9.43	4.05, 34.38	0.36, 0.81	1.29, 7.50	3.73, 32.18
P	0.65	0.002	<0.001	0.003	0.01	<0.001
Landmark 2##						
No. of patients, IVC filter/non-filter	203/658	203/658	203/658	203/658	203/658	203/658
No. of events, IVC filter/non-filter	15/37	2/9	13/7	15/37	2/9	13/7
Hazard ratio	1.80	0.33	3.43	1.55	0.33	3.32
95% CI	1.16, 2.80	0.08, 1.43	1.57, 7.53	0.97, 2.49	0.08, 1.44	1.51, 7.30
P	0.009	0.139	0.002	0.07	0.14	0.003
Landmark 3###						
No. of patients, IVC filter/non-filter	231/562	231/562	231/562	231/562	231/562	231/562
No. of events, IVC filter/non-filter	15/30	3/8	15/5	15/30	3/8	15/5
Hazard ratio	1.36	0.43	3.92	1.16	0.43	3.75
95% CI	0.84, 2.20	0.13, 1.47	1.76, 8.75	0.70, 1.95	0.12, 1.45	1.68, 8.36
P	0.21	0.18	0.001	0.56	0.17	0.001

Note: Dead or discharged at/before landmark were excluded. Univariate and multivariable Cox regression analysis.

95% CI: 95% confidence interval; IVC filter: inferior vena cava filter.

^#^Person-time before IVC filter placement (immortal time) excluded.

^##^Landmark set at day 2 after injury.

^###^Landmark set at day 3 after injury.

^*^Adjusted for age, ISS, GCS score, systolic BP and O_2_ saturation.

^**^Adjusted for age and ISS.

### Inferior vena cava filters and hazards of venous thromboembolism

IVC filter placement was not associated with PE at landmark 2 (Hazard ratio=0.33; 95% CI 0.08, 1.44; P=0.14) or landmark 3 (HR=0.43; 95% CI 0.12, 1.45; P=0.17) (Table 3). However, when person-time before IVC filter placement was excluded, IVC filter placement was associated with increased PE (HR=3.11; 95% CI 1.29, 7.50; P=0.01).

IVC filter placement was associated with increased DVT at landmark 2 (HR=3.32; 95% CI 1.51, 7.30; P=0.003), landmark 3 (HR=3.75; 95% CI 1.68, 8.36; P=0.001) and also when person-time before IVC filter placement was excluded (HR=10.95; 95% CI 3.73, 32.18; P<0.001) (Table 3).

### Inferior vena cava filters and hazards of death

IVC filter placement was not associated with all-cause mortality at landmark 2 (HR=1.55; 95% CI 0.97, 2.49; P=0.07) or landmark 3 (HR=1.16; 95% CI 0.70, 1.95; P=0.56), but when person-time before IVC filter placement was excluded, IVC filter placement was associated with reduced mortality (HR=0.54; 95% CI 0.36, 0.81; P=0.003) (Table 3).

## Discussion

The major finding in this study was that the use of IVC filters in severely injured patients without a recent history of VTE was not associated with a lower rate of PE or death. However, in patients with IVC filters inserted, the rate of DVT was higher than in those without filters.

The finding of no reduction in rates of death or PE following IVC filter placement supports the findings in a recent randomized trial demonstrating that early prophylactic placement of IVC filters in trauma patients did not lower the risk of PE or mortality at 90 days.^8^ The trial randomized 240 severely injured patients (ISS>15) with contraindication to pharmacological thromboprophylaxis to receive an IVC filter or not within 72 h after admission. Pharmacological thromboprophylaxis was, however, initiated within seven days after injury in 67% of the patients enrolled in the study. In the present study, both anticoagulation and IVC filters were used, and therefore, the data may be difficult to compare between studies.

Other observational studies have also failed to demonstrate survival benefit of prophylactic IVC filter placement in trauma patients, and IVC filters were associated with an increased risk of DVT.^16–18^ These studies used a logistic regression model to compare outcomes between the groups.^16,17^ The present study used a Cox proportional hazards model to compare events across the groups and has the ability to account for censored observations.^19^ Patients were censored at discharge date if no event had occurred.

In other indications for IVC filter placement, such as preoperative filter placement in patients with multiple risk factors for VTE undergoing bariatric surgery, studies have also failed to demonstrate that IVC filters reduce PE-related mortality.^20^ In patients with a recent history of VTE, regardless of the cause, two randomized trials have failed to show survival benefit of IVC filters.^21,22^

Both hospitals in the study used pharmacological thromboprophylaxis, in line with current guidelines.^3,23^ Due to the risk of VTE, pharmacological thromboprophylaxis is recommended for immobilized trauma patients once the risk of trauma-related bleedings is considered low.^24^ Early initiation of pharmacological thromboprophylaxis is essential and has shown to be safe.^25,26^ Even in traumatic brain injury, pharmacological thromboprophylaxis initiation 24 to 48 h postinjury has been reported to be safe.^4^

The strength of our study was the large number of patients reviewed from two large trauma centres. The sample size of 951 was larger than in the randomized trials and may better reflect the real-world practice. By using a Norwegian hospital as an external control group, we assume that the distribution of the unobserved confounder to be more similar between the control hospital and the intervention hospital than to using a local control group.^27^ When using a local control group, there is a higher risk of unobserved confounding at individual level, and the unobserved confounder is a relatively strong predictor of treatment assignment. In addition to controlling selection bias with covariate adjustment, our analyses also address immortality bias which always should be considered when exposure status is determined based on an event occurring after baseline.^7,15,28^ The present study used the Landmark approach, which is one of the available methods to reduce immortal time bias.^15^

Our study was observational and retrospective. Therefore, there are several limitations due to the study design. Due to a low number of events, a limited number of predictor variables could be included in a multivariable Cox regression analysis, although some papers have addressed that the rule of 10 events per variable might be too strict and can be relaxed.^29,30^ Because none of the patients at Oslo University Hospital Ullevål were considered for or had an IVC filter placed, it was not possible to use a propensity score for adjustment, as this would violate the strong ignorability assumption.^31^

The ISS, AIS and GCS scores reflect the injury severity status of the patient upon admission. IVC filter patients had higher ISS and AIS severity scores for chest, abdomen, bones and spines in addition to higher pulse rate and lower systolic blood pressure, but lower head AIS severity scores compared to non-IVC filter patients. The ISS is a combined effect of multi-trauma based on the anatomical AIS severity score and is calculated as the sum of the squares of the highest AIS code in each of the three most severely injured body regions. Therefore, these variables are not independent, and we considered it sufficient to include ISS and not AIS for adjustment in the model with mortality as outcome.

Because of the low number of PE and DVT events, the models using these outcomes were only adjusted for ISS and age, not for GCS score, systolic blood pressure or O_2_ saturation, which may have had an impact on the results. As the patients in the IVC filter cohort in general had more severe injuries and a higher ISS score than non-IVC filter patients, they may have been more immobile and have a higher risk of VTE, which may have influenced the results.

Shortage of variables for adjustment as well as unmeasured variables may result in residual confounding. Obesity is a known risk factor for VTE, but a variable for body mass index was not available in this study.^32^ Information on when pharmacological thromboprophylaxis was started, its duration and dosage was also not available. Additionally, there was no routine surveillance programme for VTE during the time period the study was conducted. However, surveillance programmes may increase the awareness of VTE and may detect more subclinical DVTs, but may not necessarily improve clinical outcomes by reducing PE.^33^ The present study used time to in-hospital events or death. These outcomes may be influenced by the timing of hospital discharge, and a standardized follow-up period of 30 or 90 days may have been preferable. However, most of the events would occur during the hospital stay.

As a consequence of conflicting guidelines and changing evidences, there is considerable variation in the practice of prophylactic IVC filter placement between hospitals, which cannot be explained by the underlying characteristics of the patients.^34^ The practice of prophylactic IVC filter placement is endorsed by the Eastern Association for the Surgery of Trauma in high-risk trauma patients who cannot be anticoagulated, but these guidelines have not been updated since 2002.^35,36^ In contrast, recently updated guidelines from the Society for Interventional Radiology recommend against the routine placement of IVC filters for primary prophylaxis.^37^ The results of the present study support the recommendations in the latest guidelines.

In conclusion, this study did not find that IVC filter placement was associated with a reduced rate of PE or mortality in severely injured patients. However, an increased rate of DVT was observed in patients with IVC filters inserted.

## Research ethics and patient consent

This study was approved by the Institutional Review Board (IRB) at Mayo Clinic (Ref. 12-004470), The Norwegian Regional Committee (REK) for medical and health research ethics (Ref. 2012/2293-1) and Oslo University Hospital data protection officer (Ref. 2012/18862). The large number of patients needed for this study precluded informed consent.

**Table:** 

Author name	Substantial contribution	Drafting	Final approvement	Agreement to be accountable
Thien Trung Tran	X	X	X	X
Haraldur Bjarnason	X	X	X	X
Jennifer McDonald	X	X	X	X
Nils Oddvar Skaga	X	X	X	X
Damon E Houghton	X	X	X	X
Brian Kim	X	X	X	X
Knut Stavem	X	X	X	X
Nils Einar Kløw	X	X	X	X
